# The Natural History of Antibiotic-Treated Lower Limb Cellulitis: Analysis of Data Extracted From a Multicenter Clinical Trial

**DOI:** 10.1093/ofid/ofad488

**Published:** 2023-09-29

**Authors:** O Martin Williams, Fergus Hamilton, Richard Brindle

**Affiliations:** UK Health Security Agency Microbiology Laboratory Services Bristol, Bristol Royal Infirmary, Bristol, UK; University Hospitals and Weston NHS Foundation Trust, Bristol Royal Infirmary, Bristol, UK; MRC Integrative Epidemiology Unit, University of Bristol, Bristol, UK; Infection Sciences, North Bristol NHS Trust, Bristol, UK; School of Clinical Services, University of Bristol, Bristol, UK

**Keywords:** cellulitis, leg, lower limb, natural history

## Abstract

**Background:**

Although cellulitis is a relatively common skin infection, there remains uncertainty about management, particularly the length and route of antimicrobials required. Further information on the symptomatology and biomarker changes associated with cellulitis over time would guide clinicians and patients as to the expected natural history.

**Methods:**

We extracted data from a randomized clinical trial (NCT01876628) of clindamycin as adjunctive therapy in cellulitis to illustrate the evolution of local parameters (pain, swelling, local erythema, and warmth) and the resolution of biomarkers over time.

**Results:**

Data from 247 individuals with mild to moderate unilateral lower limb cellulitis, who attended at least 1 face-to-face interview following recruitment, were used to examine response dynamics. Although there was a local improvement in swelling, warmth, erythema, and pain by day 5 compared with baseline, some individuals still had evidence of local inflammation at 10 days. Most biomarkers demonstrated a return to normal by day 3, although the initial fall in albumin only returned to baseline by day 10.

**Conclusions:**

Although there was initial resolution, a significant number of individuals still had local symptoms persisting to day 10 and beyond. Clinicians can use these data to reassure themselves and their patients that ongoing local symptoms and signs after completion of antibiotic treatment do not indicate treatment failure or warrant extension of the initial antibiotic treatment or a change in antibiotic class or mode of administration.

## Teaser

Using data routinely collected during a clinical trial we describe the natural history of antibiotic-treated lower limb cellulitis. Importantly, a significant number of individuals still have local symptoms that persist up to 10 days and beyond.

Cellulitis is a common skin infection, with an incidence ranging from 0.2/1000 person-years to 24.6/1000 person-years [1–4]. The majority of cases are uncomplicated and can be safely managed in the community setting, although the number of individuals hospitalized for cellulitis has increased over time, resulting in significant health care expenditure [5].

Published guidelines for the treatment of cellulitis [6–10] are mostly based on evidence extrapolated from studies of skin and soft tissue infections, which have included cellulitis, or are based on expert opinion. Of note, a recent systematic review of treatment guidelines of acute infections commonly seen in primary care highlighted that none of the identified guidelines for the management of cellulitis or nonpurulent skin and skin structure infections included details on the natural history of the disease [11]. Treatment decisions are complicated by the persistence of the local bacterial toxin effects and resultant local inflammation [7, 12], which do not necessarily correlate with organism load or need for prolongation of antimicrobial therapy.

Approximately 20% of individuals with cellulitis “fail” initial antimicrobial therapy [13] and are prescribed repeat courses of antimicrobials or re-attend health care. It is likely that a proportion of these individuals simply have slowly resolving symptoms and that these “failures” represent the natural history of disease. This is also complicated by guidance suggesting re-evaluation of individuals with cellulitis at 48–72 hours, leading to an assumption that patients should be improving at this point [8]. The situation is further complicated as cellulitis can be difficult to diagnose, with recognition of an increasing number of cellulitis-mimics, termed pseudo-cellulitis. Indeed, between 30% and 74% of individuals initially diagnosed and/or treated for cellulitis have an alternative final diagnosis, such as venous stasis dermatitis, contact dermatitis, eczema, lymphedema, or erythema migrans [14–16].

However, there remain limited data on the trajectory of symptoms and biomarker responses to guide patient and clinician expectations. We therefore used data routinely collected from a double-blinded placebo-controlled trial to illustrate the clinical course and response dynamics of individuals with treated mild to moderate lower limb cellulitis.

## METHODS

Data were extracted from a randomized clinical trial (NCT01876628) of antibiotic therapy for cellulitis from 20 hospitals in England. Individuals with a diagnosis of lower limb cellulitis who attended at least 1 follow-up appointment were included in this analysis. Those with cellulitis of the upper limb were excluded as this site of infection was less common and less severe, and individuals were less likely to re-attend for review [17].

Diagnosis of cellulitis was supported using criteria established for the PATCH trial on the prevention of recurrent cellulitis [18]. All adults with unilateral limb cellulitis were eligible for recruitment, but those with obvious abscesses were excluded. In addition, those who had received antibiotic treatment for >48 hours before recruitment were not included. All recruited participants were given flucloxacillin orally or intravenously, but the dose and route of administration were decided by the clinical team treating the patient. Participants on other antibiotics before recruitment were switched to flucloxacillin.

The methods of assessment have been previously reported [17]. In brief, participants had 3 face-to-face study visits scheduled: baseline (day of recruitment), day 5 (a median of 4 days after baseline), day 10 (a median of 9 days after baseline). At each visit, the affected skin area of the limb was estimated using a scoring system similar to the Psoriasis Area and Severity Index [19] from which the percentage of body skin area involved was calculated. Limb circumference was recorded at its greatest over the affected area using a disposable tape measure, and the highest temperature from the affected area was measured using an infrared thermometer [20]. Local observations of the affected limb were compared with the unaffected limb, if available. Pain scores were measured using a visual analogue scale (0–10). In addition, at each visit blood samples were taken for a full blood count, renal and liver function, and C-reactive protein. As we have previously demonstrated in this population that the addition of short-course clindamycin to flucloxacillin did not alter the clinical outcome [17] and that the route of administration (oral vs intravenous) and duration of treatment (5 days or >5 days) were not associated with recovery [21], we have combined the data from all of the individuals into 1 data set.

Statistical analysis was done using GraphPad Prism, version 9.5.0, for Windows (GraphPad Software, San Diego, CA, USA). For comparisons of variables at the 3 assessment time points, a mixed-effects analysis with Geisser-Greenhouse correction was performed. Tukey's multiple comparison test with individual variances comparing the mean of each data set was calculated for each comparison. For comparisons of the affected and unaffected limbs, an ordinary 2-way analysis of variance using a main affect model only was performed. A *P* value <.05 was considered significant. Trend line analyses were fitted using nonlinear regression with a centered third-order polynomial (cubic) equation. Due to small numbers (<20 data points), data for day 1 were not included for the patient characteristics, and data for both day 1 and day 2 were not included for blood analytes.

## RESULTS

The baseline characteristics of 247 individuals with lower limb cellulitis who attended at least 1 follow-up appointment are shown in Table 1. The majority of individuals (93.1%) had an acute presentation, with local symptoms being present for <7 days at recruitment, half of whom had been symptomatic for <2 days. In addition, at the time of recruitment 58.3% of individuals had received <12 hours of antibiotic treatment. Where the information was recorded, the median duration of antibiotic treatment following recruitment (interquartile range [IQR]) was 7 (7–9.5) days (n = 185, 74.9%); 71.5% received only oral therapy.

**Table 1.** Baseline Characteristics of 247 Patients With Lower Limb Cellulitis

Total (n = 247)		Normal Range
Age, mean (SD), y	52.2 (18.0)	…
Male, No. (%)	166 (67.2)	…
Duration of local features before recruitment, mean (SD), d	3.5 (6.9)	…
Duration of flucloxacillin before recruitment, mean (SD), d	0.5 (0.6)	…
Affected skin area as percentage of body surface area, median (IQR)	6.0 (4.0–8.0)	…
Difference in circumference between affected and unaffected limbs, median (IQR), cm	2.1 (1.1–4.1)	…
Difference in surface temperature between affected and unaffected limbs, median (IQR), °C	2.3 (1.2–3.6)	…
Pain score (0–10), median (IQR)	5 (3–7)	…
Hemoglobin, median (IQR), g/L	136 (122–145)	130–170
Neutrophil, median (IQR), ×10^9^/L	6.7 (4.5–9.4)	1.5–8.0
Lymphocyte, median (IQR), ×10^9^/L	1.5 (1.2–2.0)	1.0–4.0
Neutrophil/lymphocyte ratio, median (IQR)	4.2 (2.5–7.3)	…
Platelets, median (IQR), ×10^9^/L	222 (179–269)	150–400
Urea, median (IQR), mmol/L	5.1 (4.2–6.6)	2.5–7.8
Creatinine, median (IQR), μmol/L	79 (67–96)	59–104
Albumin, median (IQR), g/L	38 (34–42)	35–50
C-reactive protein, median (IQR), mg/L	43.5 (13.2–115.8)	<6.0
Alkaline phosphatase, median (IQR), U/L	78 (63–97)	30–130
Alanine transaminase, median (IQR), U/L	23 (18–35)	10–50

Abbreviation: IQR, interquartile range.

**Figure 1.**
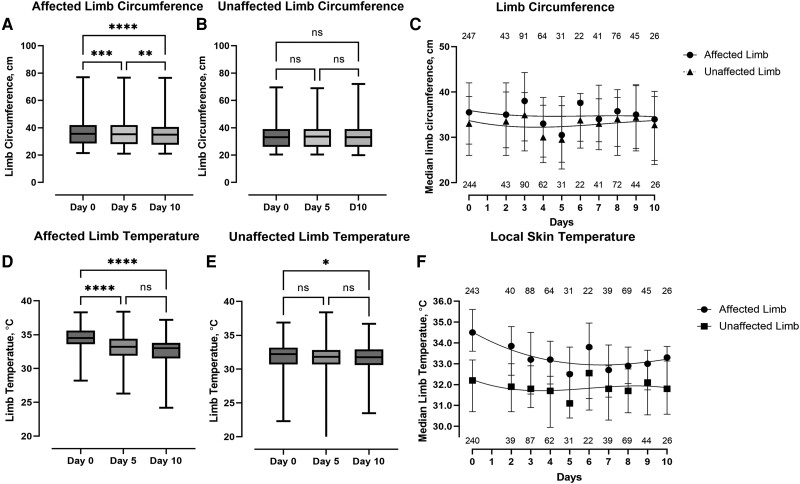
Box plots showing median, IQR, and maximum/minimum values. *A* and *B*, The circumference of the affected and unaffected lower limbs at baseline and at the day 5 and day 10 assessments, respectively. *D* and *E*, The local temperature of the affected and unaffected lower limbs at baseline and at the day 5 and day 10 assessments, respectively. ****P < .0001; ***P < .001; **P < .01; *P < .05. *C* and *F* Trend lines of the median and IQR of the circumference (*C*) and the local temperature (*F*) of the affected and unaffected limbs from baseline to day 10. The numbers above and below the lines represent the number of observations included at each time point. Data from day 1 have been omitted due to small numbers (n < 20). Abbreviation: IQR, interquartile range; ns, non-signficant.

Overall, there was a reduction in mean limb circumference of the affected limb between baseline and day 5 (mean reduction, 0.5 cm; 95% CI, 0.17–0.81; *P* = .001), baseline and day 10 (mean reduction, 0.85 cm; 95% CI, 0.45–1.25; *P* < .001), and between the day 5 and day 10 assessments (mean reduction, 0.36 cm; 95% CI, 0.08–0.63; *P* = .0072) (Figure 1*A–C*). Despite the reduction in edema by day 10, there remained a difference in the mean circumference between the affected and unaffected limbs (mean difference, 1.94 cm; 95% CI, 1.60–2.28; *P* < .0001), with more than a third (36.4%) of individuals having a ≥2-cm difference in circumference between the affected and unaffected limbs. There was a similar reduction in the mean temperature of the affected limb between baseline and day 5 (mean reduction, 1.4°C; 95% CI, 1.0–1.8; *P* < .001) and between baseline and day 10 (mean reduction, 1.8°C; 95% CI, 1.4–2.2; *P* < .0001) (Figure 1*D–F*). Despite the reduction in local warmth by day 10, there remained a significant difference in the mean local temperature between the affected and unaffected limbs (mean difference, 1.1°C; 95% CI, 0.9–1.3; *P* < .0001). Again, approximately one-third of individuals (29.5%) had ≥1°C greater difference in local skin temperature between the affected and unaffected limbs at day 10.

**Figure 2.**
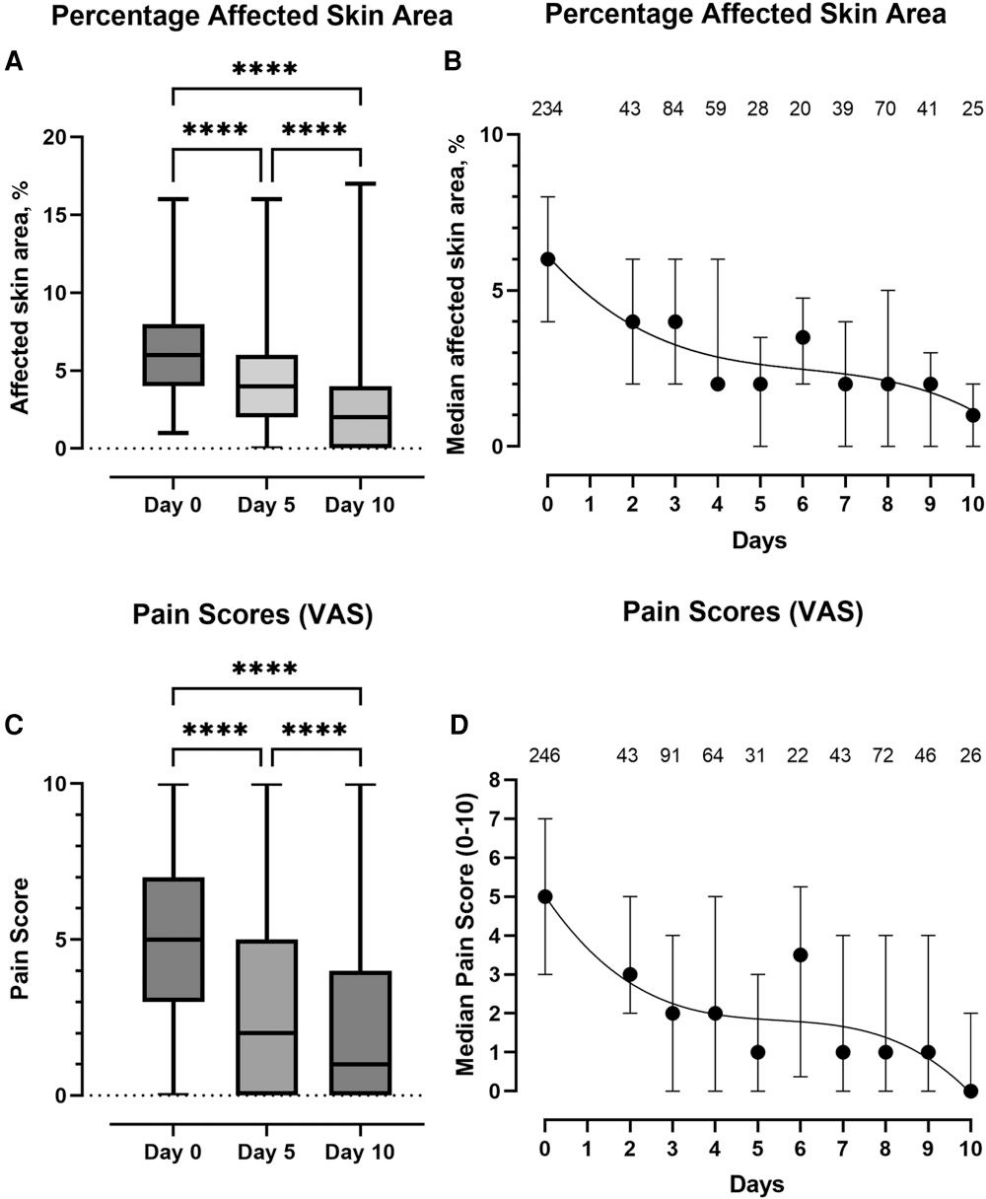
Box plots showing median, IQR, and maximum/minimum values. *A*, The percentage of affected skin area at baseline and at the day 5 and day 10 assessments, respectively. *C*, Pain scores at baseline and at the day 5 and day 10 assessments, respectively. ****P < .0001; ***P < .001; **P < .01; *P < .05. *B* and *D*, Trend lines of the median and IQR of the percentage of affected skin area (B) and the local temperature (*D*) of the affected limb from baseline to day 10. The numbers above the lines represent the number of observations included at each time point. Data from day 1 have been omitted due to small numbers (n < 20). Abbreviation: IQR, interquartile range.

There was a 34% reduction in the mean percentage of affected surface area between baseline and day 5 (*P* < .0001), and a further reduction of 31.1% between day 5 and day 10 (*P* < .0001), with an overall reduction in the mean percentage of affected skin area of 54.8% (*P* < .0001) (Figure 2*A* and *B*). In addition to a reduction in local swelling, warmth, and the area of affected skin, there was also a significant reduction in local pain between baseline and day 5 (mean reduction, 2.0; 95% CI, 1.6–2.4; *P* < .0001), baseline and day 10 (mean reduction, 2.9; 95% CI, 2.5–3.3; *P* < .0001), and day 5 and day 10 (mean reduction, 0.9; 95% CI, 0.6–1.2; *P* < .0001), although more than half of individuals (54.3%) continued to report a pain score of ≥1, and 13.9% reported a pain score of ≥5 on day 10 (Figure 2*C* and *D*).

**Figure 3.**
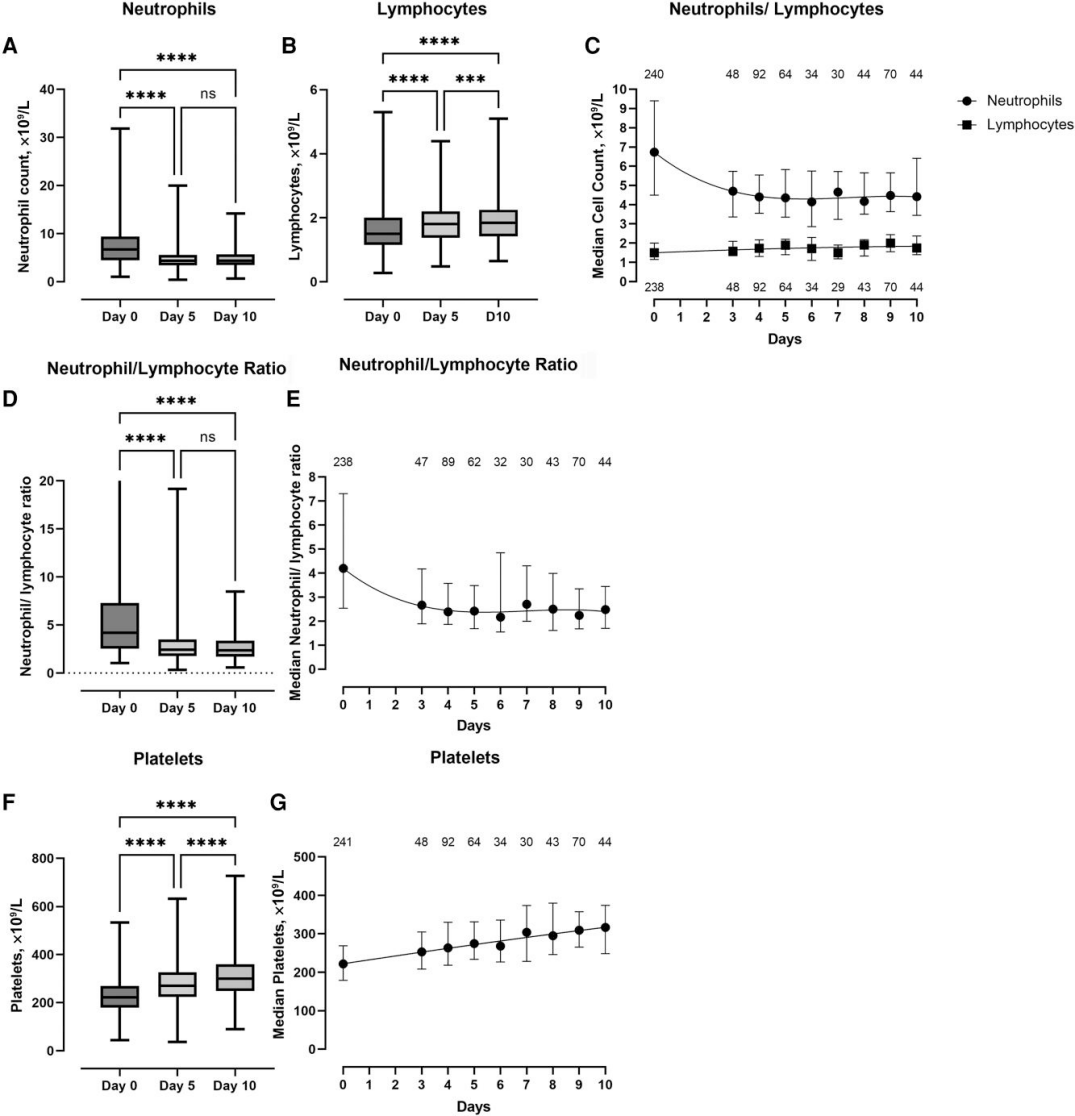
Box plots showing median, IQR, and maximum/minimum values. *A*, *B*, *D*, and *F*, The neutrophil count, lymphocyte count, NLR, and platelet count at baseline and at the day 5 and day 10 assessments, respectively. ****P < .0001; ***P < .001; **P < .01; *P < .05. *C*, The daily trend of the median and IQR of the neutrophil and lymphocyte counts. *E* and *G*, Trends for NLR and platelet count, respectively, to day 10. The numbers above and below the lines represent the number of observations included at each time point. Data from day 1 and day 2 have been omitted due to small numbers (n < 20). Abbreviations: IQR, interquartile range; NLR, neutrophil/lymphocyte ratio; ns, non-significant.

**Figure 4.**
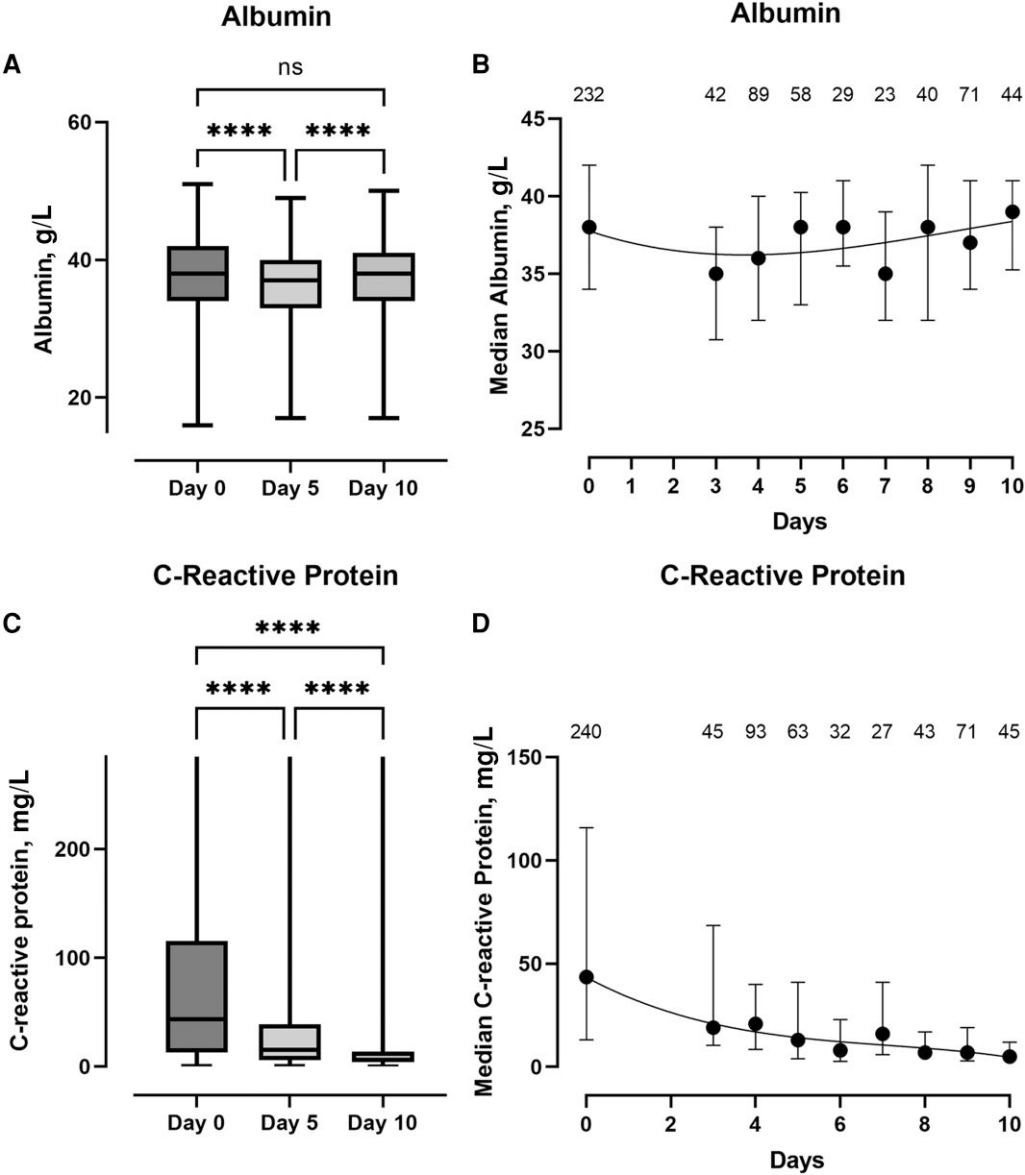
Box plots showing median, IQR, and maximum/minimum values. *A* and *C*, Albumin and C-reactive protein measurements at baseline and at the day 5 and day 10 assessments, respectively. ****P < .0001; ***P < .001; **P < .01; *P < .05. *B* and *D*, The trends of the median and IQR of albumin and C-reactive protein, respectively, from baseline to day 10. The numbers above the lines represent the number of observations included at each time point. Data from day 1 and 2 have been omitted due to small numbers (n < 20). Abbreviation: IQR, interquartile range; ns, non-significant.

When comparing the various hematological and biochemical parameters, there was a reduction in the total neutrophil count between baseline and day 5, as well as baseline and day 10 (Figure 3*A*), appearing to stabilize by day 3 (Figure 3*C*), with an associated recovery in the total lymphocyte count between baseline and day 5, and between day 5 and day 10 (Figure 3*B* and *C*). In parallel, there was a significant reduction in the neutrophil/lymphocyte ratio (NLR) between baseline and day 5, and baseline and day 10 (Figure 3*D*), appearing to stabilize from day 3 (Figure 3*E*). In addition, there was a significant increase in the platelet count between baseline and day 5, and day 5 and day 10 (Figure 3*F* and *G*). Data on other hematological and biochemical parameters are included in the Supplementary Figures (hemoglobin, Supplementary Figure 1; urea and creatinine, Supplementary Figure 2; alkaline phosphatase and alanine transaminase, Supplementary Figure 3). There was a small but significant drop in albumin between the baseline measurement and day 5, with a trend toward recovery by day 10 (Figure 4*A* and *B*). Finally, baseline C-reactive protein (CRP) was significantly elevated, with significant falls at day 5 and a return toward normal levels by day 10 (Figure 4*C* and *D*).

## DISCUSSION

Lower limb cellulitis remains a common infection syndrome, with the greatest numbers occurring predominantly in the summer months [4, 5, 22]. It causes a significant drain on health resources [23, 24], but there remains uncertainty about the most appropriate form of therapy, and little in the literature documents the natural history of antibiotic-treated disease.

The data presented here, derived from a clinical trial on the antibiotic management of mild to moderate lower limb cellulitis, demonstrate that although there are early responses in laboratory parameters, there is a delay in the resolution of local symptoms and signs. This has a number of implications for clinicians and patients. First, clinicians and patients should expect prolonged symptomatology, and early re-assessment should be considered only in light of worsening, not ongoing, symptoms. Second, the use of “rescue” antimicrobials should again be limited to those with worsening symptoms. Finally, these data should inform the conduct and outcome of randomized trials of therapies to alleviate cellulitis. There have been a number of randomized controlled trials on the antibiotic treatment of cellulitis (see a recent meta-analysis and systematic review [25]). The outcomes of these clinical trials have been the subject of a systematic review [26], which demonstrated the lack of consensus on the description of outcomes, with most trials using the terms “cure, improvement, success, failure.” There was also a lack of consensus for the time point for assessment of clinical response, with some trials measuring outcomes at several time points ranging from 48 hours to 42 days.

In 2013, the US Food and Drug Administration (FDA) recommended that the primary efficacy end point for antibiotic clinical trials of acute bacterial skin and skin structure infections (ABSSSIs) be based on a percent reduction (≥20%) in lesion size at 48–72 hours compared with baseline, with complete resolution at 7–14 days after completion of therapy as a secondary end point [27]. This was based in part on the observations by Snodgrass and Anderson [28]. Early assessment of clinical response was tentatively supported by a recent meta-analysis [29], although it has been criticized by others [30] as early responses to treatment are not the primary aim of treatment. In contrast, recently updated European guidelines continue to support the assessment of the primary end point at the test of cure visit, after completion of therapy [31].

Our data support a more nuanced assessment of outcomes in cellulitis, which should include patient-reported outcomes (eg, pain and symptomatology), as these extend for longer than previously reported. The need for the use of more patient-focused and patient-reported outcomes in clinical trials on cellulitis has been identified in a recent systematic review [26], as they reflect the real day-to-day impact on patients' lives. Additionally, given the extended symptomatology, these data support consideration of adjunctive therapies to reduce inflammation (such as corticosteroids [32] or nonsteroidal anti-inflammatory drugs [33]), as antimicrobial therapy will be ineffective at improving symptoms. Further trials on the effectiveness, cost-effectiveness, and safety of adjunctive therapies in cellulitis are required.

Our data are in line with the previous literature, although we generally identified greater and longer symptomatology. A recent study of 216 hospitalized patients with cellulitis examined both early and late clinical responses after initiation of antibacterial treatment, including both clinical and biochemical parameters [34]. Of note, ∼11% of recruited patients had an associated wound or ulcer. At day 1 of treatment, it was reported that 55% of cases had cessation of local spread, and 52% had improvement of local inflammation (defined as the intensity of erythema, warmth, and tenderness), with a combination of the 2 occurring in 39%. Defervescence (defined as a body temperature ≤37.5°C in ≥2 separate measurements among cases with temperature >37.5°C the day before) was recorded in <40% of cases. By day 2, ∼90% of cases had cessation of local spread, with 88% having improvement in local inflammation, with the combination of the 2 occurring in 85%. Despite the local improvement, nearly 40% had failed to defervesce. Finally, by day 3 nearly all cases (>95%) had a cessation of local spread and improvement of local inflammation, but 15% had failed to defervesce. Of note, a lack of clinical response at day 3 was not predictive of clinical failure post-treatment. In contrast, Cranendonk and colleagues in the DANCE trail described ongoing patient-reported local symptoms (pain) and investigator-assessed signs (edema and cellulitis severity score) up until day 14 and beyond, with only an estimated 75% reduction in symptom scores at day 14 compared with day 1 [35].

Yadav and colleagues undertook a systematic review and meta-analysis of randomized controlled trials on antibiotic treatment of uncomplicated cellulitis where data on the time to clinical response of a variety of clinical parameters were included [29]. Although outcomes were inconsistently defined, the overall pooled median time to clinical response was 1.68 days (95% CI, 1.48–1.88; 4 studies). A 50% reduction in pain (4 studies) and severity (3 studies) scores was identified by day 5 of treatment, with a gradual resolution in pain and severity reported over 2–4 weeks. There was an approximately one-third reduction in redness diameter by day 2–3 of treatment, and a further one-third reduction by day 7–14 (2 studies). Finally, there was a 30%–50% reduction in local edema by day 2–4 of treatment (3 studies), and 50%–85% by day 7–10.

Our analysis has a number of strengths. First, data were collected prospectively within a randomized trial, with minimal loss to follow-up and detailed assessment of a number of parameters for a prolonged period of time. Second, the inclusion criteria of our trial were pragmatic, unlike many other trials in skin and soft tissue infection. Limitations of our post hoc secondary data analysis include those of the original randomized trial; it is likely that loss to follow-up was not random and patients with greater (or fewer) symptoms may have been more likely to have follow-up. Additionally, trial populations are known to be “healthier” on average than the reference population in which the trial is run. This would suggest, however, that our reported results are an underestimate of the burden of symptoms of cellulitis, although they do highlight that even those considered to have mild infection can still suffer with prolonged symptoms. Finally, given the recognized difficulty in clinically diagnosing cellulitis, it is possible that individuals with pseudocellulitis may have been recruited. Although this could bias the overall duration of symptoms, it is likely that the number of individuals with cellulitis mimics was small. The pragmatic design of the trial reflects the experiences of real life, and clinicians and patients need to understand that ongoing symptoms beyond the period of active antibiotic treatment are not unusual and do not indicate treatment failure.

## CONCLUSIONS

The natural history of cellulitis is long, with symptoms present in many patients more than a week after diagnosis. Clinicians should inform patients of this natural history and should limit repeat assessment and antimicrobial therapy to those with worsening, not continuing, symptoms. Additional research is required to investigate the benefits of adjunctive therapies, but any future trials on cellulitis need to include patient-focused outcomes with an extended duration of follow-up beyond 10 days to better understand the natural history of the disease.

## Supplementary Material

ofad488_Supplementary_Data

## Supplementary Data

Supplementary materials are available at *Open Forum Infectious Diseases* online. Consisting of data provided by the authors to benefit the reader, the posted materials are not copyedited and are the sole responsibility of the authors, so questions or comments should be addressed to the corresponding author.
