# Supplementary material for: The Natural History of Antibiotic-Treated Lower Limb Cellulitis: Analysis of Data Extracted From a Multicenter Clinical Trial
Source: Open Forum Infect Dis. 2023 Sep 29;10(10):ofad488. doi: 10.1093/ofid/ofad488 (PMC10578506; doi:10.1093/ofid/ofad488)
Supplement: ofad488_Supplementary_Data [file ofad488_supplementary_data.docx]

Supplementary Material

Additional blood analytes

Index

Page 2. Figure S1: Haemoglobin

Page 3. Figure S2: Urea and creatinine

Page 4. Figure S3: Alkaline phosphatase and alanine transaminase


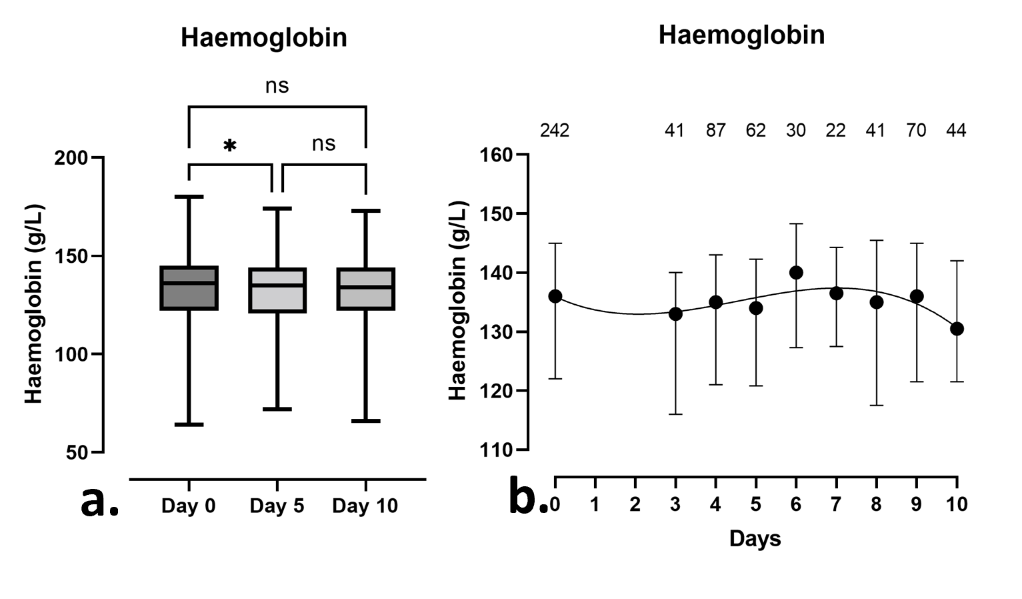


Figure S1. Box plots showing median, IQR and maximum/ minimum values. Figure S1a, show haemoglobin levels at baseline and at the day 5 and day 10 assessments, respectively. *=p<0.05. Figures S1b shows the trend lines for haemoglobin from baseline to day 10. The numbers above line represent the number of observations included at each time point. Data from day 1 and day 2 have been omitted due to small numbers (n<20).


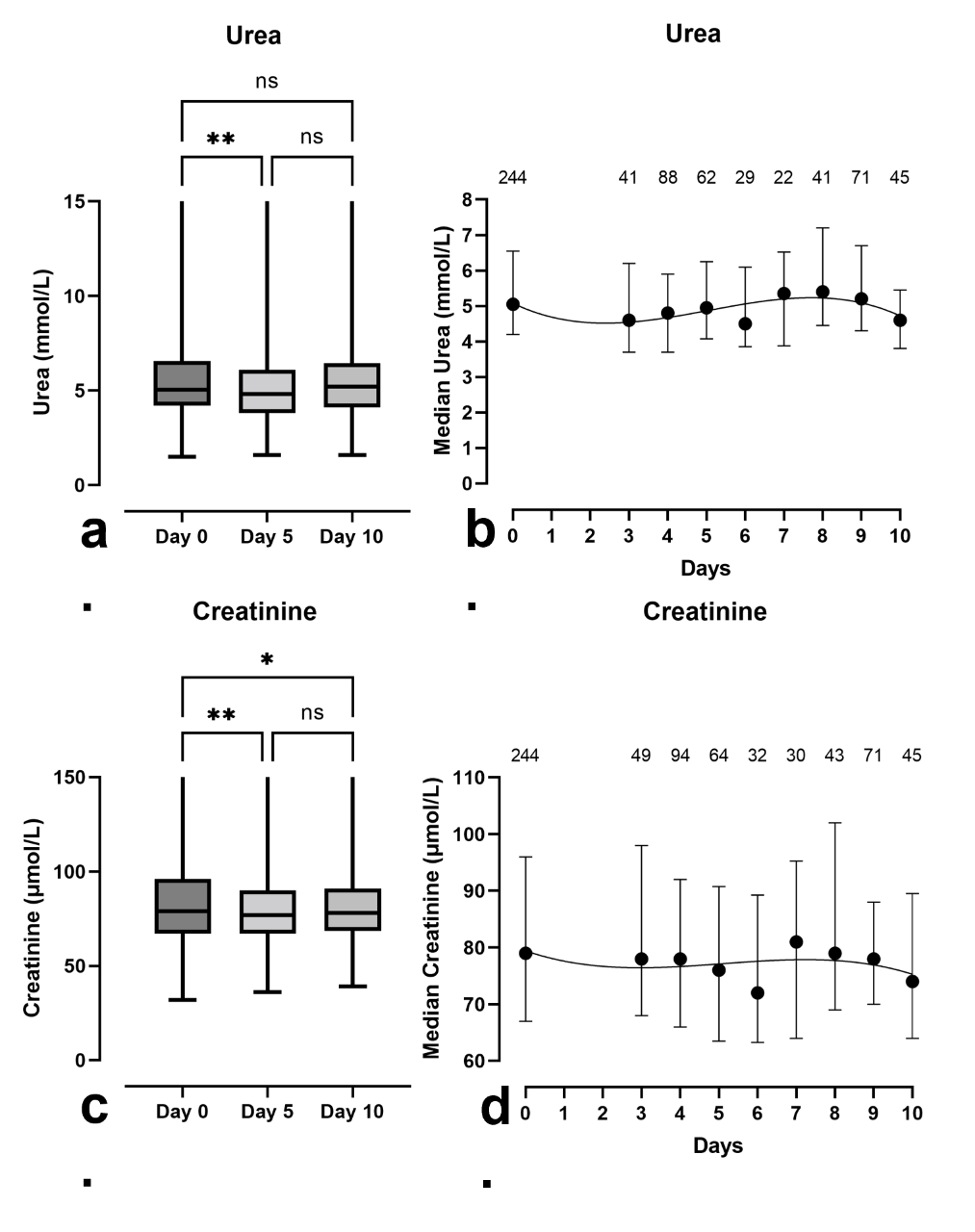


Figure S2. Box plots showing median, IQR and maximum/ minimum values. Figure S2a, and S2c show the urea and creatinine at baseline and at the day 5 and day 10 assessments, respectively. **=p<0.01; *=p<0.05. Figure S2b and S2d show trend lines of the median and IQR of the urea and creatinine, respectively, from baseline to day 10. The numbers above the lines represent the number of observations included at each time point. Data from day 1 and day 2 have been omitted due to small numbers (n<20).


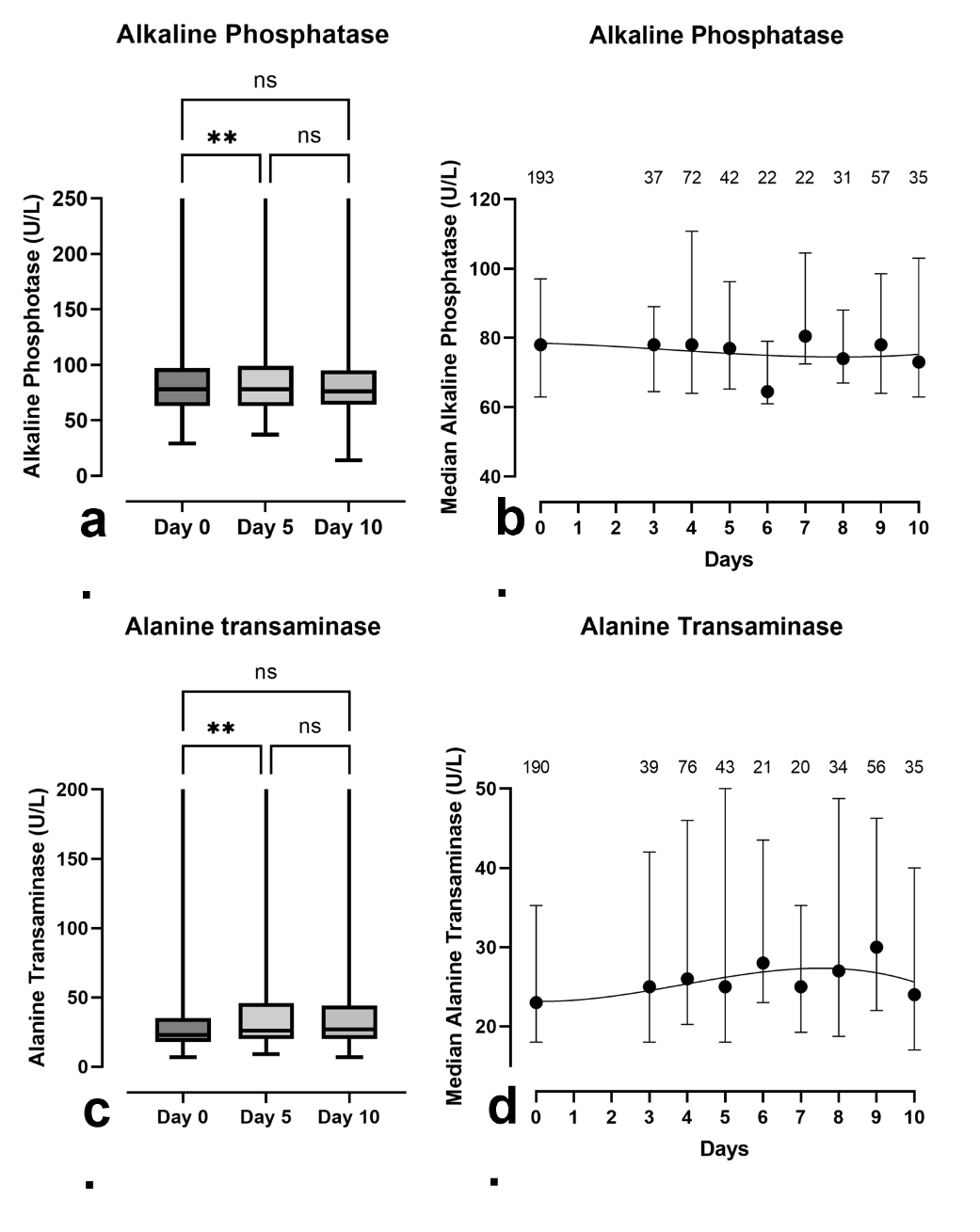


Figure S3. Box plots showing median, IQR and maximum/ minimum values. Figure S3a, and S3c show the alkaline phosphatase and alanine transaminase at baseline and at the day 5 and day 10 assessments, respectively. **=p<0.01. Figures S3b and S3d show trend lines of the median and IQR of alkaline phosphatase and alanine transaminase from baseline to day 10. The numbers above the lines represent the number of observations included at each time point. Data from day 1 and day 2 have been omitted due to small numbers (n<20)
